# Development of a Lentivirus Vector-Based Assay for Non-Destructive Monitoring of Cell Fusion Activity

**DOI:** 10.1371/journal.pone.0102433

**Published:** 2014-07-16

**Authors:** Zeinab Neshati, Jia Liu, Guangqian Zhou, Martin J. Schalij, Antoine A. F. de Vries

**Affiliations:** 1 Heart Center Leiden, Leiden University Medical Center, Leiden, The Netherlands; 2 Shenzhen University Medical School, Shenzhen University, Shenzhen, People’s Republic of China; University of Minnesota Medical School, United States of America

## Abstract

Cell-to-cell fusion can be quantified by endowing acceptor and donor cells with latent reporter genes/proteins and activators of these genes/proteins, respectively. One way to accomplish this goal is by using a bipartite lentivirus vector (LV)-based cell fusion assay system in which the cellular fusion partners are transduced with a flippase-activatable *Photinus pyralis luciferase* (*PpLuc*) expression unit (acceptor cells) or with a recombinant gene encoding FLPe^NLS+^, a nuclear-targeted and molecularly evolved version of flippase (donor cells). Fusion of both cell populations will lead to the FLPe-dependent generation of a functional *PpLuc* gene. PpLuc activity is typically measured in cell lysates, precluding consecutive analysis of one cell culture. Therefore, in this study the PpLuc-coding sequence was replaced by that of *Gaussia princeps* luciferase (GpLuc), a secretory protein allowing repeated analysis of the same cell culture. In myotubes the spread of FLPe^NLS+^ may be limited due to its nuclear localization signal (NLS) causing low signal outputs. To test this hypothesis, myoblasts were transduced with LVs encoding either FLPe^NLS+^ or an NLS-less version of FLPe (FLPe^NLS−^) and subsequently co-cultured in different ratios with myoblasts containing the FLPe-activatable *GpLuc* expression cassette. At different times after induction of cell-to-cell fusion the GpLuc activity in the culture medium was determined. FLPe^NLS+^ and FLPe^NLS−^ both activated the latent *GpLuc* gene but when the percentage of *FLPe*-expressing myoblasts was limiting, FLPe^NLS+^ generally yielded slightly higher signals than FLPe^NLS−^ while at low acceptor-to-donor cell ratios FLPe^NLS−^ was usually superior. The ability of FLPe^NLS+^ to spread through myofibers and to induce reporter gene expression is thus not limited by its NLS. However, at high FLPe concentrations the presence of the NLS negatively affected reporter gene expression. In summary, a rapid and simple chemiluminescence assay for quantifying cell-to-cell fusion progression based on GpLuc has been developed.

## Introduction

During cell-to-cell fusion, plasma membranes of individual cells merge to form a multinucleated structure called a syncytium. Plasma membrane fusion is a crucial event during, for example, fertilization, syncytiotrophoblast production, skeletal muscle formation, bone remodeling, eye lens development and certain forms of tissue repair [1]. In general, cell fusion is a tightly regulated and highly selective process involving specific cell types. Inappropriate cell fusion has been implicated in tumor development and progression [2].

Cell fusion can be easily observed using microscopic techniques and in many studies the extent of cell fusion is expressed as fusion index, which either stands for the percentage of cells with two or more nuclei or the percentage of nuclei present in syncytia [3]. However, without continuous monitoring, it is impossible to decide by microscopy alone whether multinucleation is caused by cell fusion or the result of karyokinesis without cytokinesis. In addition, cells growing on top of each other can be mistaken for syncytia. Furthermore, as fusion index determinations are generally carried out manually, they are laborious, error-prone and often inaccurate. This has led to the development of methods for quantifying cell fusion independent of microscopic inspection. Nearly all these methods are based on systems of two components that interact to create a novel detectable signal only after cell fusion [3]. Mohler and Blau, for example, developed a quantitative cell fusion assay based on functional complementation between two biologically inactive β-galactosidase deletion mutants [4]. Another possibility to produce fusion-dependent signals is by applying site-specific recombination systems such as Cre-loxP and FLP-FRT. In these systems, a latent reporter gene is activated by the action of the site-specific DNA recombinase Cre from bacteriophage P1 or flippase/FLP from *Saccharomyces cerevisiae*, which catalyze the excision and inversion of DNA flanked by 34-base pair (bp) recognition sequences (loxP for Cre and FRT for FLP) in a direct or inverted repeat configuration, respectively [5], [6].

Gonçalves *et al.* previously developed a bipartite lentivirus vector (LV)-based cell fusion assay system in which the cellular fusion partners are endowed with a FLP-activatable *Photinus pyralis luciferase* (*PpLuc*) expression unit/”gene switch” (acceptor cells) or with a recombinant gene encoding a molecularly evolved version of FLP (FLPe) with a nuclear localization signal (NLS) derived from the simian virus 40 large T antigen (donor cells) [7]. Fusion between acceptor and donor cells led to the FLPe-dependent generation of a functional episomal *PpLuc* expression module. This cell fusion monitoring system was successfully used to study the role of the p38 MAPK signaling pathway in myoblast fusion/myotube formation. However, since PpLuc is a cytoplasmic protein and its substrate D-luciferin is poorly membrane-permeable, this assay requires lysis of the cells prior to luminometry and does not allow repeated analysis of the same cell culture. This prompted us to develop a nondestructive method to quantify cell fusion using the bipartite LV-based cell fusion assay system described by Gonçalves and colleagues as starting point.

The key difference between the new and “old” version of the LV-based cell fusion assay system is the replacement of the *PpLuc* open reading frame (ORF) in the “original” gene switch construct by the humanized coding sequence of *Gaussia princeps* luciferase (GpLuc), which is a secretory protein converting the substrate coelenterazine into coelenteramide plus light. GpLuc also displays a much higher specific luciferase activity than PpLuc and is exceptionally resistant to exposure to heat and strongly acidic and basic conditions [8]. In addition, we hypothesized that in myotubes the spread of nuclear-targeted FLPe (FLPe^NLS+^) beyond the direct surroundings of donor nuclei may be limited due to the presence of the NLS. This would result in the activation of only a fraction of the reporter genes especially in hybrid myotubes containing a relatively low percentage of *FLPe* gene-positive donor nuclei compared to GpLuc-encoding acceptor nuclei. To test this hypothesis, we generated an LV encoding an NLS-less version of FLPe (FLPe^NLS−^) and compared, in myogenic fusion assays, its ability to activate latent *GpLuc* genes with that of FLPe^NLS+^.

## Materials and Methods

### Plasmids

DNA constructions were carried out with enzymes from Fermentas (Fisher Scientific, Landsmeer, the Netherlands) or from New England Biolabs (Bioké, Leiden, the Netherlands) by using established procedures [9] or following the instructions provided with specific reagents.

**Figure 1:**
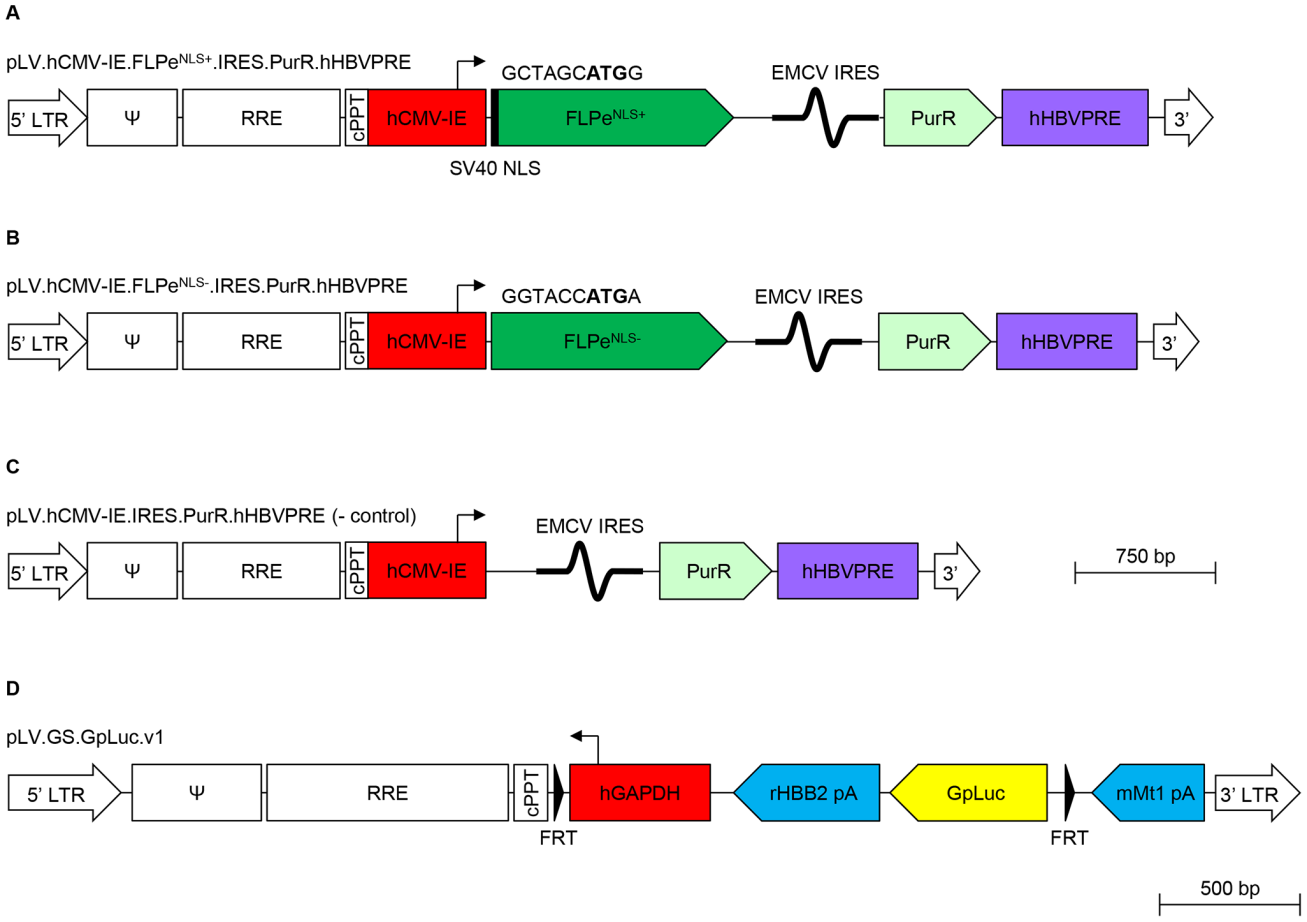
Structure of the LV DNA in the LV shuttle plasmids. (**A**): pLV.hCMV-IE.FLPe^NLS+^.IRES.PurR.hHBVPRE (**B**): pLV.hCMV-IE.FLPe^NLS−^.IRES.PurR.hHBVPRE (**C**): pLV.hCMV-IE.IRES.PurR.hHBVPRE and (**D**): pLV.GS.GpLuc.v1. The start codons of the FLPe^NLS+^ and FLPe^NLS−^ ORFs are shown in boldface. 5′ LTR, chimeric 5′ long terminal repeat containing the Rous sarcoma virus U3 region and the HIV1 R and U5 regions; Ψ, HIV1 packaging signal; RRE, HIV1 Rev-responsive element; cPPT, HIV1 central polypurine tract and termination site; hCMV-IE, human cytomegalovirus *immediate early* gene promoter; FLPe^NLS+^, molecularly evolved flippase with simian virus 40 (SV40) nuclear localization signal (NLS; black bar); FLPe^NLS−^, molecularly evolved flippase without NLS; EMCV IRES, encephalomyocarditis virus internal ribosomal entry site; PurR; *Streptomyces alboniger* puromycin N-acetyl transferase-coding sequence; hHBVPRE, human hepatitis B virus posttranscriptional regulatory element; black triangle/FRT, flippase recognition target sequence; hGAPDH, human *glyceraldehyde 3-phosphate dehydrogenase* gene promoter; rHBB2 pA, rabbit *β-hemoglobin* gene polyadenylation signal; GpLuc, *Gaussia princeps* luciferase-coding sequence; mMT1 pA, mouse *metallothionein 1* gene polyadenylation signal; 3′ LTR, 3′ HIV1 long terminal repeat containing a deletion in the U3 region to render the LV self-inactivating.

To generate a bicistronic self-inactivating (SIN) human immunodeficiency virus type 1 (HIV1) vector shuttle plasmid coding for *Streptomyces alboniger* puromycin N-acetyl transferase (PurR) and FLPe^NLS−^, pLV.FLPe.PurR ([7]; GenBank accession number: GU253314; hereinafter referred to as pLV.hCMV-IE.FLPe^NLS+^.IRES.PurR.hHBVPRE; Fig. 1A) was digested with BshT1 and Eco81I and the 9.6-kb DNA fragment containing the vector backbone was purified from agarose gel. The hybridization product of oligodeoxyribonucleotides 5′ CCGGTACCATGAGTCAATTTGATATATTATGTAAAACACCACC 3′ and 5′ TTAGGTGGTGTTTTACATAATATATCAAATTGACTCATGGTA 3′ (both from Eurofins MWG Operon, Ebersberg, Germany) was combined with the 9.6-kb BshT1×Eco81I fragment of pLV.hCMV-IE.FLPe^NLS+^.IRES.PurR.hHBVPRE by ligation with bacteriophage T4 DNA ligase producing pLV.hCMV-IE.FLPe^NLS−^.IRES.PurR.hHBVPRE (Fig. 1B).

**Figure 2:**
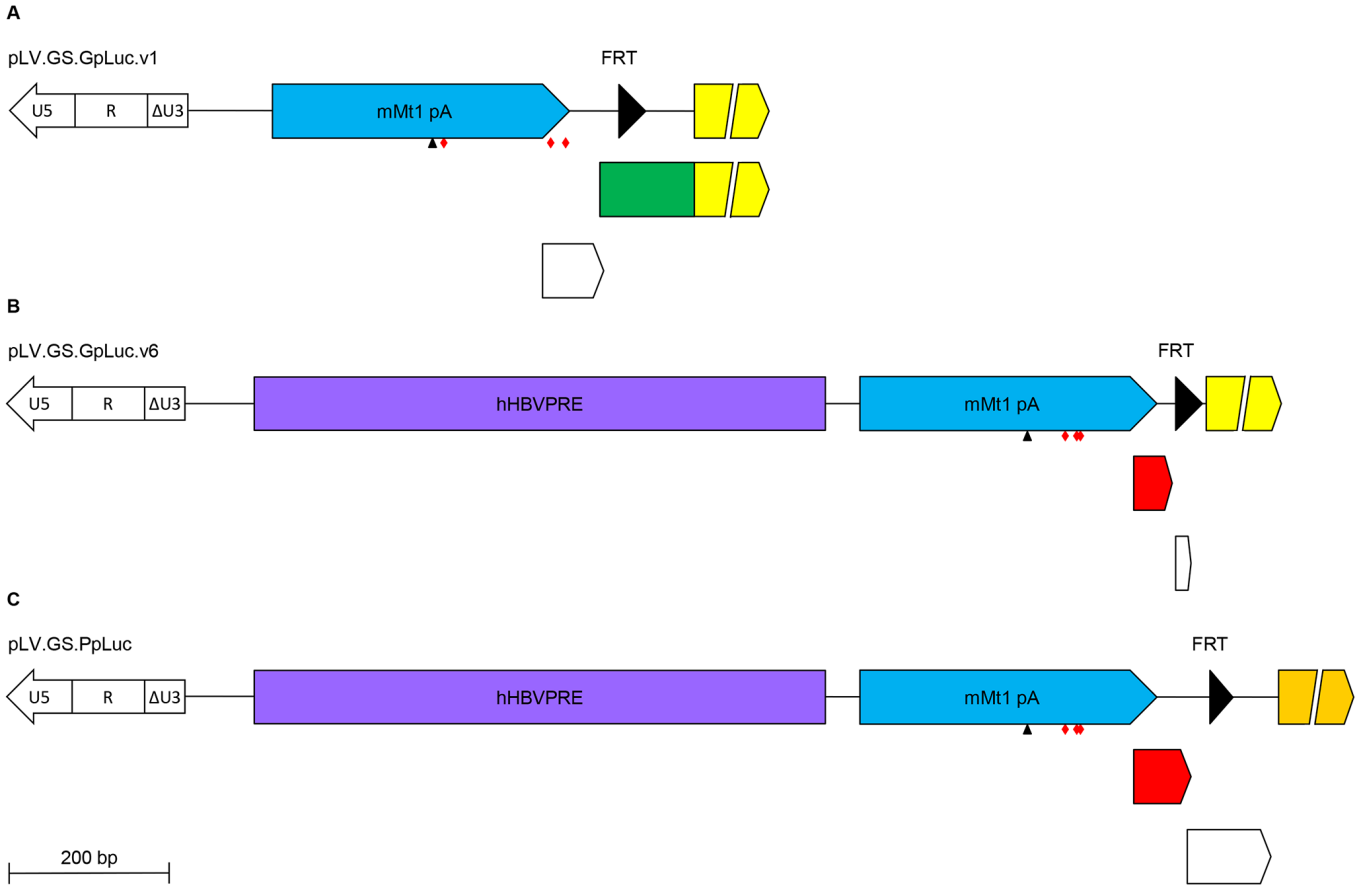
Improved design of the *GpLuc* gene switch cassette. (**A–C**): Detailed structure of the areas upstream of the *Luc* ORFs in pLV.GS.GpLuc.v1 (A), pLV.GS.GpLuc.v6 (B) and pLV.GS.PpLuc (C) starting at the HIV1 3′ LTR. U5, HIV1 LTR unique 5′ region; R, HIV1 LTR repeat region; ΔU3, enhancer- and promoterless HIV1 LTR unique 3′ region; blue arrow, mouse *metallothionein 1* gene polyadenylation signal (mMT1 pA); small black triangle, AATAAA motif in mMT1 pA; red diamonds, stop codons in frame with *Luc* ORFs; large black triangle, minimal FRT sequence; light yellow arrow, *GpLuc* ORF; green box, 5′ in-frame extension of the *GpLuc* ORF; white arrows, out-of-frame ORFs preceding *Luc* ORFs; red arrows, in-frame ORFs preceding *Luc* ORFs; dark yellow arrow, *PpLuc* ORF.

**Figure 3:**
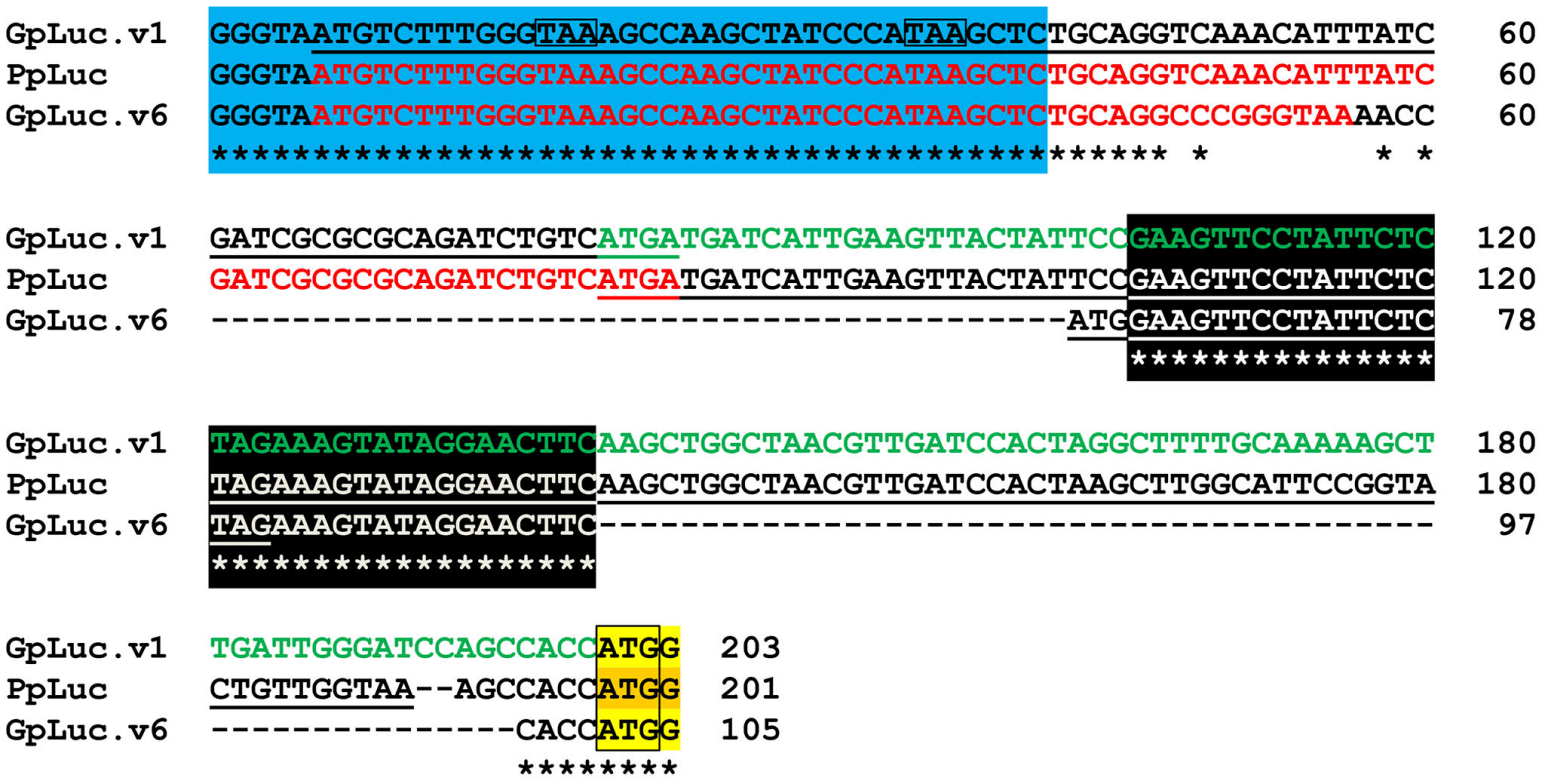
Alignment of the nucleotide sequences immediately upstream of the *Luc* ORFs in pLV.GS.GpLuc.v1, pLV.GS.PpLuc and pLV.GS.GpLuc.v6. Blue box, 3′ end of the mMT1 pA; underlined sequences, out-of-frame ORFs preceding *Luc* ORFs; boxed TAA sequences, in-frame stop codons preceding *Luc* ORFs; red letters, in-frame ORFs preceding *Luc* ORFs; green letters, 5′ in-frame extension of the *GpLuc* ORF in pLV.GS.GpLuc.v1; black box, minimal FRT sequence; boxed ATG sequences, *Luc* initiation codons; light yellow box, 5′ end *GpLuc* ORF; dark yellow box, 5′ end *PpLuc* ORF.

To generate a SIN-LV shuttle plasmid carrying a silent *GpLuc* gene that can be activated by FLP, cloning vector pR6K.MCS was digested with XmaJI and NotI, the 2.2-kb DNA fragment containing the vector backbone was purified from agarose gel and combined with the 0.6-kb GpLuc-encoding XmaJI×NotI fragment of phGluc.dBamHI yielding construct pR6K.GpLuc. The cloning vector pR6K.MCS was derived from construct pA1.GFP.A2 ([10]; GenBank accession number: GQ380658) by combining its 2.0-kb SalI×Aflll fragment with the 0.3-kb SalI×Aflll fragment of pMOLUC ([11]; Addgene, Cambridge, MA; plasmid number: 12514). Plasmid phGluc.dBamHI was made from the mammalian expression vector phGluc ([12]; Addgene; plasmid number: 22522) by self-ligation of its 2.9-kb BamHI fragment. The *GpLuc* ORF was excised from pR6K.GpLuc by digestion with XmaJI and MluI and combined with the 7.2-kb BcuI×MluI fragment of pLV.GS.DsRed.dKpnI to generate pLV.GS.GpLuc.v1 (Fig. 1D). The LV shuttle plasmid pLV.GS.DsRed.dKpnI was derived from pLV.pA+.GS.DsRed ([7]; GenBank accession number: GU253312) by self-ligation of its 7.9-kb KpnI fragment. The SIN-LV shuttle plasmid pLV.GS.GpLuc.v6 is a derivative of construct pLV.pA+.GS.Luc ([7], hereinafter referred to as pLV.GS.PpLuc), in which the sequences interspersed between the rabbit *β-hemoglobin* gene polyadenylation signal (rHBB pA) and the mouse *metallothionein 1* gene (mMT1) pA (*i.e.* the *PpLuc* ORF and an FRT sequence) are replaced by a synthetic DNA fragment comprising the *GpLuc* ORF and an FRT sequence. More details about the genetic makeup of pLV.GS.GpLuc.v1, pLV.GS.GpLuc.v6 and pLV.GS.PpLuc and about the nucleotide sequences located in between the mMT1 pA and *Luc* ORFs of these three SIN-LV plasmids are provided in Figs. 2 and 3.

The ligation mixtures were introduced in chemocompetent cells of *Escherichia coli* strain GeneHogs (Life Technologies Europe, Bleiswijk, the Netherlands) or GT115 (InvivoGen, San Diego, CA). Large-scale plasmid purifications were performed using JETSTAR 2.0 Plasmid Maxiprep kits (Genomed, Löhne, Germany) according to the manufacturer’s instructions.

### Cells

The culture and differentiation conditions of the murine Bmi1- and human TERT-immortalized human myoblasts (iDMD myoblasts) have been described previously [13].

### Viral vectors

The vesicular stomatitis virus G protein-pseudotyped SIN-LVs LV.FLPe^NLS+^.PurR, LV.FLPe^NLS−^.PurR, LV.PurR (negative control vector), LV.GS.GpLuc.v1, LV.GS.PpLuc and LV.GS.GpLuc.v6 were generated in 293T cells with the aid of the LV shuttle plasmids pLV.hCMV-IE.FLPe^NLS+^.IRES.PurR.hHBVPRE, pLV.hCMV-IE.FLPe^NLS−^.IRES.PurR.hHBVPRE, pLV.CMV.IRES.PURO ([14], hereinafter referred to as pLV.hCMV-IE.IRES.PurR.hHBVPRE; Fig. 1C), pLV.GS.GpLuc.v1, pLV.GS.PpLuc and pLV.GS.GpLuc.v6, respectively. The 293T cells were transfected with one of the LV shuttle constructs and the packaging plasmids psPAX2 (Addgene; plasmid number: 12260) and pLP/VSVG (Life Technologies Europe) at a molar ratio of 2∶1:1. To concentrate and purify the LV particles, producer cell supernatants were layered onto 5-ml cushions of 20% (wt/vol) sucrose in phosphate-buffered saline (PBS) and centrifuged at 15,000 rotations per minute for 2 h at 4°C in an SW32 rotor (Beckman Coulter Nederland, Woerden, the Netherlands). Prior to ultracentifugation, producer cell supernatants were clarified by low speed centrifugation and filtration through 0.45-µm pore-sized cellulose acetate filters (Pall Netherlands, Mijdrecht, the Netherlands). For more details about the SIN-LV production method, see [15]. The titers of the resulting LV stocks were determined using the RETROTEK HIV-1 p24 Antigen ELISA kit (ZeptoMetrix, Franklin, MA) following the instructions provided by the manufacturer. To derive functional titers from these measurements a conversion factor of 2.5 transducing units (TUs) per pg of HIV-1 p24 protein was used.

### Cell transductions

Cryopreserved LV.FLPe^NLS+^.PurR-transduced iDMD myoblasts ([7]; hereinafter referred to as myoblasts-FLPe^NLS+^) were thawed and cultured in the presence of puromycin (Life Technologies Europe) at a final concentration of 0.4 µg/ml to prevent transgene silencing. *FLPe^NLS–^*expressing iDMD myoblasts were generated by overnight (±20 h) exposure of 10^5^ cells in a well of a 24-well cell culture plate (Greiner Bio-One, Alphen aan den Rijn, the Netherlands) to 30 TUs of LV.FLPe^NLS−^.PurR per cell in 500 µl of growth medium in a humidified atmosphere of 5% CO_2_/95% air at 37°C. The next day, the cell monolayer was rinsed three times with 1 ml of PBS after which fresh culture medium was added. At 3 days post transduction, the culture of LV.FLPe^NLS−^.PurR-treated cells (hereinafter referred to as myoblasts-FLPe^NLS−^) as well as a control culture of untransduced iDMD myoblasts were given medium containing 0.8 µg/ml of puromycin. Within a week, all cells in the culture of untransduced iDMD myoblasts had died while the cells in the LV.FLPe^NLS−^.PurR-treated culture were nicely expanding. The myoblasts-FLPe^NLS−^ were passaged once a week (split ratio 1∶3) in growth medium containing 0.4 µg/ml of puromycin. Myoblasts^GS.GLuc^, myoblasts^GS.PLuc^ and myoblasts^GS.GLuc+^ were generated likewise by exposure of iDMD myoblasts to LV.GS.GpLuc.v1, LV.GS.PpLuc and LV.GS.GpLuc.v6, respectively. Before being used for co-culture experiments, the cells were passaged at least three times to rule out secondary transduction of the *FLPe*-expressing myoblasts in the co-cultures with luciferase-encoding SIN-LVs [16].

### Co-culture establishment and maintenance

Co-cultures containing a total number of 2×10^5^ cells were established in wells of 24-well culture plates by mixing myoblasts-FLPe^NLS+^ or myoblasts-FLPe^NLS−^ with myoblasts^GS.GLuc^ at the indicated ratios. Following an incubation period of about 72 h when the cell monolayers had reached 90–100% confluency, the growth medium was substituted by 400 µl of either differentiation medium or fresh growth medium. At specified time points thereafter, the culture medium (400 µl) was collected and stored at −80°C for luciferase assay. The co-cultures were then either terminated or further incubated at 37°C in a water-saturated atmosphere of 5% CO_2_/95% air.

To compare the performance of the newly developed LV.GS.GpLuc.v1-based cell fusion assay system with that of the previously described LV.GS.PpLuc-based cell fusion quantification method [7], myoblasts^GS.GLuc^ or myoblasts^GS.PLuc^ were co-cultured with myoblasts-FLPe^NLS+^ in different ratios in 24-well culture plates containing 2×10^5^ cells per well. Samples (culture fluid for cultures containing myoblasts^GS.GLuc^ and cell lysates for cultures containing myoblasts^GS.PLuc^) were harvested 96 h and 120 h after induction of myogenic differentiation. Exactly the same approach was used to compare the LV.GS.GpLuc.v1- and LV.GS.GpLuc.v6-based cell fusion assays.

### Immunocytology

At different time points after the initiation of differentiation, 1∶1 co-cultures of myoblasts-FLPe^NLS−^ and myoblasts^GS.GLuc^ were fixed by incubation for 30 minutes at room temperature (RT) in PBS containing 4% formaldehyde. To permeabilize the cells, they were exposed for 10 minutes at RT to 0.1% Triton X-100 in PBS. Next, cells were incubated overnight at 4°C with mouse anti-skeletal muscle troponin I (skTnI) primary antibody (HyTest, Turku, Finland; clone 12F10) diluted 1∶100 in PBS +0.1% donkey serum (Santa Cruz Biotechnology, Santa Cruz, CA) followed by a 2-h incubation at RT with Alexa Fluor 568-conjugated donkey anti-mouse IgG (H+L) secondary antibody (Life Technologies Europe) diluted 1∶400 in PBS +0.1% donkey serum. Counterstaining of nuclei was performed with 10 µg/ml Hoechst 33342 (Life Technologies Europe) in PBS. Cells were washed three times with PBS after fixation, permeabilization and incubation with primary antibody, secondary antibody and DNA-binding fluorochrome. To minimize photobleaching, coverslips were mounted in Vectashield mounting medium (Vector Laboratories, Burlingame, CA). Pictures were taken with a fluorescence microscope equipped with a digital color camera (Nikon Eclipse 80i; Nikon Instruments Europe, Amstelveen, the Netherlands) using NIS Elements software (Nikon Instruments Europe).

### Subcellular fractionation and western blotting

Myoblasts-FLPe^NLS+^ and myoblasts-FLPe^NLS−^ were cultured separately in 24-well cell culture plates at a density of 2×10^5^ cells per well. Following an incubation period of 72 h when the cell monolayers had reached 90–100% confluency, the growth medium was substituted by 400 µl of either differentiation medium or fresh growth medium. Ninety-six h later, cell fractionation was carried out as described by Suzuki *et al.* [17] with the following modifications. Cell pellets were suspended in 97.5 µl of ice-cold 0.1% NP40 in PBS. One-third of the lysate was removed as “whole cell lysate” and mixed with 5 µl of 10× NuPAGE Sample Reducing Agent and 12.5 µl of 4× NuPAGE LDS Sample Buffer (both from Life Technologies Europe). The rest of the lysate was briefly centrifuged at 4°C after which 32.5 µl of the supernatant was removed as “cytosolic fraction” and supplemented with 5 µl of 10× NuPAGE Sample Reducing Agent and 12.5 µl of 4× NuPAGE LDS Sample Buffer. The remaining supernatant was removed and the pellet was washed with and suspended in 30 µl PBS, after which 5 µl of 10× NuPAGE Sample Reducing Agent and 12.5 µl of 4× NuPAGE LDS Sample Buffer were added to produce the “nuclear fraction”. Nuclear fractions and whole cell lysates were sonicated for 2 times 10 seconds at 200 Hz using a Soniprep 150 ultrasonic disintegrator (Measuring and Scientific Equipment, London, United Kingdom). After incubating the samples for 1 minute at 100°C, 10 µl of whole cell lysate, 10 µl of cytosolic fraction and 5 µl of nuclear fraction were applied to a NuPAGE Novex 12% Bis-Tris gel (Life Technologies Europe). Following electrophoretic separation, the proteins were transferred to a polyvinylidene difluoride membrane (Amersham Hybond P; GE Healthcare Europe, Diegem, Belgium) by wet electroblotting. Next, the membrane was incubated with 2% ECL AdvanceTM blocking agent (GE Healthcare Europe) in PBS-0.1% Tween 20 (PBST) for 1 h at RT and probed with rabbit anti-FLP (1∶200; Diagenode, Seraing, Belgium; CS-169–100), mouse anti-glyceraldehyde 3-phosphate dehydrogenase (GAPDH; 1∶10,000; Merck Millipore, Billerica, MA; clone 6C5) or rabbit anti-lamin A/C (1∶10,000; Santa Cruz Biotechnology; sc-20681) primary antibodies overnight at 4°C, followed by a 1-h incubation with appropriate horseradish peroxidase-conjugated secondary antibodies (Santa Cruz Biotechnology). GAPDH served as cytoplasmic marker protein and lamin A/C antibody was used as nuclear marker protein. Target protein signals were visualized using the SuperSignal West Femto Maximum Sensitivity Substrate Kit (Thermo Scientific, Rockford, IL) and chemiluminescence was measured with the ChemiDoc XRS imaging system (Bio-Rad Laboratories, Veenendaal, the Netherlands).

### FLPe functionality test

To test the functionality of the FLPe molecules encoded by LV.FLPe^NLS+^.PurR and LV.FLPe^NLS−^.PurR, myoblasts^GS.GLuc^ were transduced with LV.FLPe^NLS+^.PurR, LV.FLPe^NLS−^.PurR or LV.PurR. Myoblasts^GS.GLuc^ were seeded in a 24-well cell culture plate at a density of 10^5^ cells per well and exposed for 20 h to 75 µl per well of concentrated vector stock diluted in growth medium to a final volume of 500 µl. Next, the cell monolayers were rinsed three times with 1 ml of PBS after which 400 µl fresh growth medium was added. At 24 h after the removal of the inoculum, the culture medium was collected and transiently stored at −80°C for subsequent analysis of luciferase activity. The cells were overlaid with 400 µl of fresh growth medium, which was harvested 24 h later for storage at −80°C until luciferase activity measurement.

### Luciferase assay

After thawing the GpLuc-containing samples on ice, 50 µl of each sample was transferred to a well of a white opaque 96-well flat-bottom microtiter plate (OptiPlate-96; PerkinElmer, Groningen, the Netherlands) for chemiluminescence measurements. The native coelenterazine (Promega Benelux, Leiden, the Netherlands) stock solution (5 mg/ml in acidified methanol) was diluted 1,000 times in phenol red-free Dulbecco’s modified Eagle’s medium (Life Technologies Europe) and equilibrated for 1 h in the dark at RT before starting the measurements. The luciferase activity was measured at RT with the aid of a Wallace 1420 VICTOR 3 multilabel plate reader with automatic injection system (PerkinElmer). Immediately after automated addition of 20 µl of substrate to a well, substrate and sample were mixed by shaking for 1 second (double orbital, 0.1 mm, normal speed). PpLuc activity was measured in cell lysates as previously described [7]. For each condition, three independent samples were measured in three series of measurements.

### Statistical analysis

Different experimental groups were compared using the independent samples *t*-test. Differences among means were considered significant at *P*≤0.05. Graphs were prepared in GraphPad Prism version 5 (GraphPad Software, La Jolla, CA).

## Results

### Microscopic analysis of cell fusion kinetics

**Figure 4:**
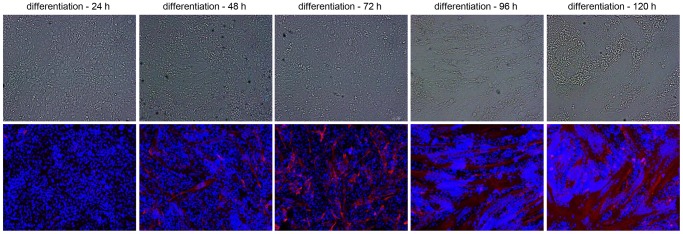
Microscopic analysis of cell fusion kinetics in 1∶1 co-cultures of myoblasts-FLPe^NLS^^−^ and myoblasts^GS.GLuc^ after maintenance for 72 h in growth medium and subsequent exposure to differentiation medium. At 24, 48, 72, 96 or 120 h after initiation of differentiation the cells were fixed and immunostained for skTnI (red fluorescence). The blue fluorescence corresponds to nuclei labeled with the karyophilic dye Hoechst 33342. The upper and lower row of pictures show phase-contrast images and fluoromicrographs, respectively. The first syncytia appeared at ±48 h after serum removal. The cell cultures displayed a time-dependent increase in frequency and size of myotubes/sacs until the cells started to detach from the surface of the culture plates. In parallel cultures of myoblasts kept in normal growth medium the cells remained firmly attached to their support and only few small syncytia were observed at late times after culture initiation (data no shown).

Cultured myoblasts can be prompted to fuse with each other by withdrawing mitogens from the culture medium. This causes a time-dependent accumulation of nuclei in syncytial structures called myotubes or myosacs depending on whether these structures are elongated or rounded. To get a first impression of the cell-to-cell fusion kinetics of the genetically modified iDMD myoblasts, 1∶1 co-cultures of myoblasts-FLPe^NLS−^ and myoblasts^GS.GLuc^ were exposed to myogenic differentiation conditions. As shown in the upper panel of Fig. 4, the myoblasts started to fuse 48 h after serum withdrawal resulting in the formation of myotubes/sacs. Both the percentage of nuclei present in myotubes/sacs as well as the size of the syncytia increased with time until 120 h following serum removal, after which the cells started to detach from the surface of the culture plates. The fusion process was accompanied by the accumulation of sarcomeric proteins as evinced by the results of the skTnI-specific immunostaining depicted in the lower panel of Fig. 4.

### Immunodetection of FLPe in LV.FLPe^NLS+/−^.PurR-transduced iDMD myoblasts

**Figure 5:**
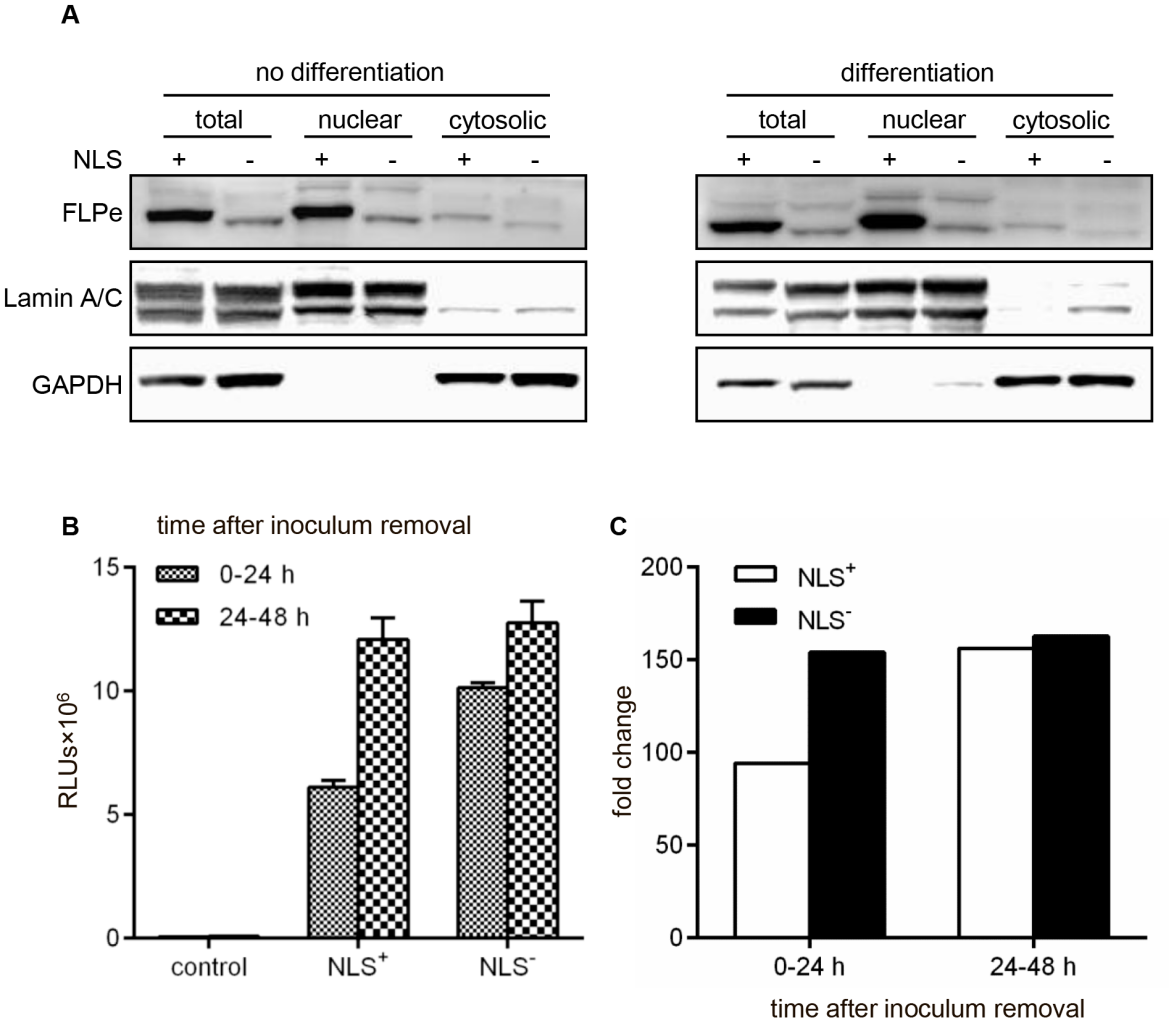
Analysis of FLPe^NLS+/−^ level, intracellular localization and enzymatic activity. (**A**): Western blotting analysis of whole protein lysates, nuclear cell fractions and cytosolic cell fractions of myoblasts-FLPe^NLS+^ (+) and of myoblasts-FLPe^NLS−^ (−) maintained in growth medium (no differentiation) or exposed to differentiation conditions for 96 h (differentiation). (**B**): Luciferase activity measurements in culture media of myoblasts^GS.GLuc^ transduced with LV.FLPe^NLS+^.PurR, LV.FLPe^NLS−^.PurR or LV.PurR (negative control vector) representing different intervals (*i.e.* 0–24 h and 24–48 h) post transduction. Bars show mean ± standard error of the mean (n = 3). (**C**): Fold change in luciferase activity calculated on the basis of the data presented in (B). The average light production by samples of LV.PurR-transduced myoblasts^GS.GLuc^ was the denominator and the mean of the RLUs produced by LV.FLPe^NLS+^.PurR-transduced myoblasts^GS.GLuc^ (NLS^+^) or by LV.FLPe^NLS−^.PurR-transduced myoblasts^GS.GLuc^ (NLS^−^) was the numerator. NLS, nuclear localization signal; FLPe, molecularly evolved flippase; GAPDH, glyceraldehyde 3-phosphate dehydrogenase; RLUs, relative light units.

To compare FLPe protein level and intracellular distribution between myoblasts-FLPe^NLS+^ and myoblasts-FLPe^NLS−^, western blot analysis was performed on whole cell lysates as well as on nuclear and cytosolic cell fractions (Fig. 5A). As expected from the presence at its amino terminus of the SV40 NLS, FLPe^NLS+^ (predicted molecular weight: 49.7 kilodaltons) had a slightly lower gel mobility than FLPe^NLS−^ (predicted molecular weight: 48.6 kilodaltons). Both under growth and differentiation conditions, the steady-state level of FLPe^NLS+^ was considerably higher than that of FLPe^NLS−^ even though the nucleotide sequences upstream of the FLPe start codon are very similar and both proteins contain a “destabilizing” amino acid residue (serine in FLPe^NLS−^ versus alanine in FLPe^NLS+^; [18]) immediately downstream of the initiator methionine. Fig. 5A also reveals that a larger fraction of FLPe^NLS+^ molecules than of FLPe^NLS−^ molecules resides in the nucleus (nuclear-to-cytosolic ratios under differentiation conditions of 8.4 and 3.1, respectively) consistent with the presence in FLPe^NLS+^ of an SV40 NLS.

### Assessment of FLPe^NLS+/−^ functionality

**Figure 6:**
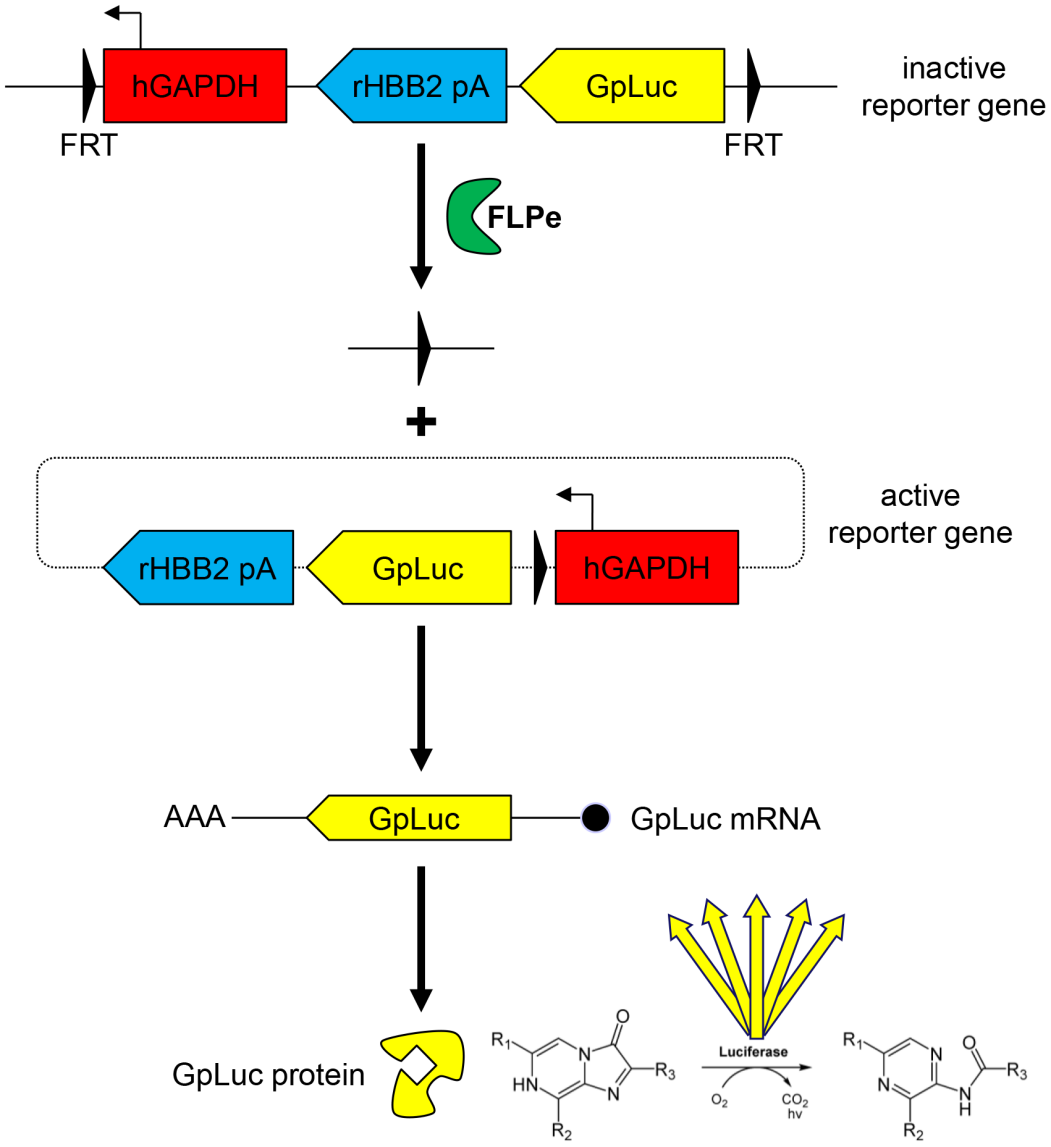
Schematic overview of the activation of the *GpLuc* gene switch cassette. Recognition of the FRT sites in chromosomally integrated copies of the LV.GS.GpLuc genome by FLPe leads to the activation of the latent *GpLuc* gene through the formation of circular episomes positioning the *hGAPDH* gene promoter upstream of the *GpLuc* ORF. Black triangle/FRT, flippase recognition target sequence; hGAPDH, human *glyceraldehyde 3-phosphate dehydrogenase* gene promoter; rHBB2 pA, rabbit *β-hemoglobin* gene polyadenylation signal; GpLuc, *Gaussia princeps* luciferase-coding sequence; FLPe, molecularly evolved flippase.

To investigate the functionality of the FLPe molecules encoded by LV.FLPe^NLS+^.PurR and LV.FLPe^NLS−^.PurR, myoblasts^GS.GLuc^ were transduced with either of these FLPe-encoding SIN-LVs or with LV.PurR (negative control vector). Production of functional recombinases by the FLPe-encoding SIN-LVs should result in activation of the *GpLuc* gene switch cassettes incorporated into the genomes of the myoblasts^GS.GLuc^ and the secretion of active GpLuc molecules in their culture medium (Fig. 6). Analysis of the culture media harvested at 24 h after vector removal showed strong luciferase activity in the samples derived from the LV.FLPe^NLS+^.PurR- and LV.FLPe^NLS−^.PurR-transduced myoblasts^GS.GLuc^, while hardly any luciferase activity was detected in the culture medium of LV.PurR-transduced myoblasts^GS.GLuc^ (Fig. 5B). During the next 24-h interval the luciferase activity in the culture media of LV.FLPe^NLS+^.PurR- and LV.FLPe^NLS−^.PurR-transduced myoblasts^GS.GLuc^ further increased whereas the luciferase activity in the negative control samples remained very low. As a result, luciferase activity was 94/154- and 156/162-fold higher in 0–24 h and 24–48 h culture medium of LV.FLPe^NLS+^.PurR- and LV.FLPe^NLS−^.PurR-transduced myoblasts^GS.GLuc^, respectively, than in the corresponding culture media of LV.PurR-infected cells (Fig. 5C). These findings confirm the presence of FLP recombinase-activatable *GpLuc* expression units in myoblasts^GS.GLuc^ and demonstrate that LV.FLPe^NLS+^.PurR and LV.FLPe^NLS−^.PurR both code for functional FLPe molecules.

### Validation of the LV.FLPe^NLS+/−^.PurR/LV.GS.GpLuc-based cell fusion assay system

**Figure 7:**
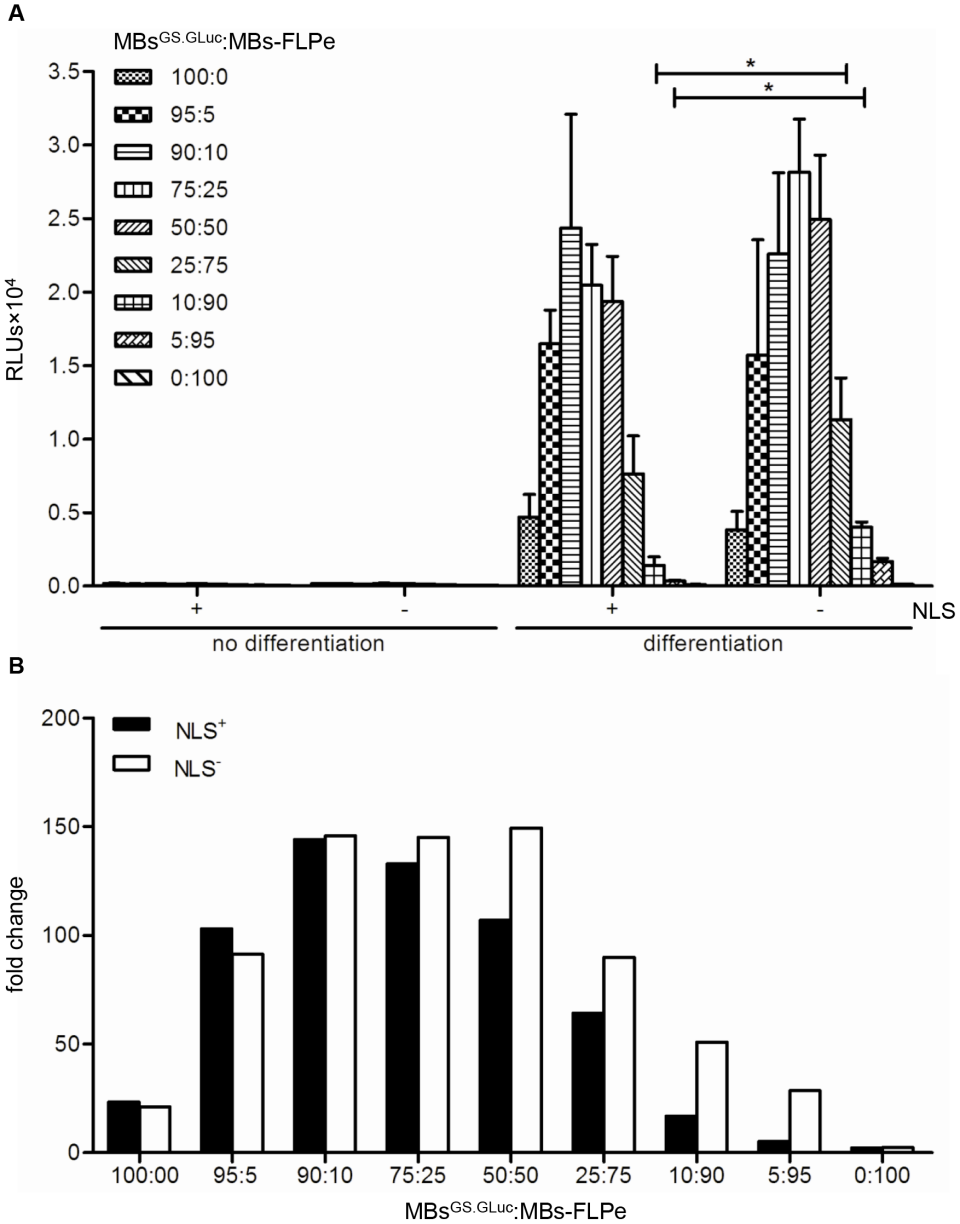
Validation of the LV.FLPe^NLS+/−^.PurR/LV.GS.GpLuc.v1-based cell fusion assay system. (**A**): Luminometric analysis of culture medium of myoblasts^GS.GLuc^ co-cultured with myoblasts-FLPe^NLS+^ (+) or with myoblasts-FLPe^NLS−^ (−) at the indicated ratios. At 72 h after cell seeding, the culture fluid in each well was replaced by fresh culture medium with (growth conditions, no differentiation) or without (differentiation conditions) serum. Nighty-six h later the culture media were collected and subjected to luciferase activity measurements. Bars represent mean ± standard error of the mean (n = 3). (**B**): Fold change in luciferase activity calculated on the basis of the data presented in (A). For each culture composition the average light production under growth conditions was the denominator and the mean of the RLUs produced under differentiation conditions was the numerator. RLUs, relative light units; MBs^GS.GLuc^, myoblasts^GS.GLuc^; MBs-FLPe, myoblasts-FLPe; NLS, nuclear localization signal.

To compare the ability of FLPe^NLS+^ and FLPe^NLS−^ to activate the *GpLuc* gene switch upon cell fusion, myoblasts^GS.GLuc^ were co-cultured with myoblasts-FLPe^NLS+^ or myoblasts-FLPe^NLS−^ at different ratios (*i.e.* 95∶5, 90∶10, 75∶25, 50∶50, 25∶75, 10∶90 and 5∶95). Monocultures of myoblasts-FLPe^NLS+^, myoblasts-FLPe^NLS−^ or myoblasts^GS.GLuc^ exposed to growth or differentiation medium and co-cultures of *FLPe*-expressing myoblasts and myoblasts^GS.GLuc^ maintained in growth medium served as negative controls. Based on the results of the microscopic analysis of cell fusion activity (Fig. 4), the culture medium was harvested 96 h after induction of myogenic differentiation. It should be noted, however, that the kinetics of cell fusion progression slightly differed between individual experiments probably reflecting small differences in the myoblast populations used for different experiments. Luciferase activity in the medium of the fusogenic cell cultures depended on the ratio of myoblasts^GS.GLuc^ and myoblasts-FLPe, showed a similar trend for myoblasts-FLPe^NLS+^- and myoblasts-FLPe^NLS–^containing co-cultures and was highest when co-cultures contained 50–95% myoblasts^GS.GLuc^ (Fig. 7A). The peak of GpLuc activity was reached at myoblast^GS.GLuc^:myoblast-FLPe ratios of 90∶10 and 75∶25 for myoblasts-FLPe^NLS+^ and myoblasts-FLPe^NLS−^, respectively (Fig. 7A). Interestingly, at low myoblast^GS.GLuc^:myoblast-FLPe ratios (*i.e.* 10∶90 and 5∶95) the luciferase activity was significantly higher for myoblasts-FLPe^NLS−^ than for myoblasts-FLPe^NLS+^ (Fig. 7A). Myoblast cultures kept under growth conditions and myoblast-FLPe monocultures maintained in differentiation medium yielded luminescence signals close to or at background levels. The monocultures of myoblasts^GS.GLuc^ did, however, secrete detectable amounts of GpLuc under differentiation conditions although the signal intensity was much lower than that produced by serum-deprived co-cultures containing 50–90% myoblasts^GS.GLuc^. For the co-cultures containing 50–90% myoblasts^GS.GLuc^ shifting from growth to differentiation medium resulted in a >100-fold increase in luciferase activity (Fig. 7B).

### Use of the LV.FLPe^NLS+/−^.PurR/LV.GS.GpLuc-based cell fusion assay system to analyse cell fusion progression

**Figure 8:**
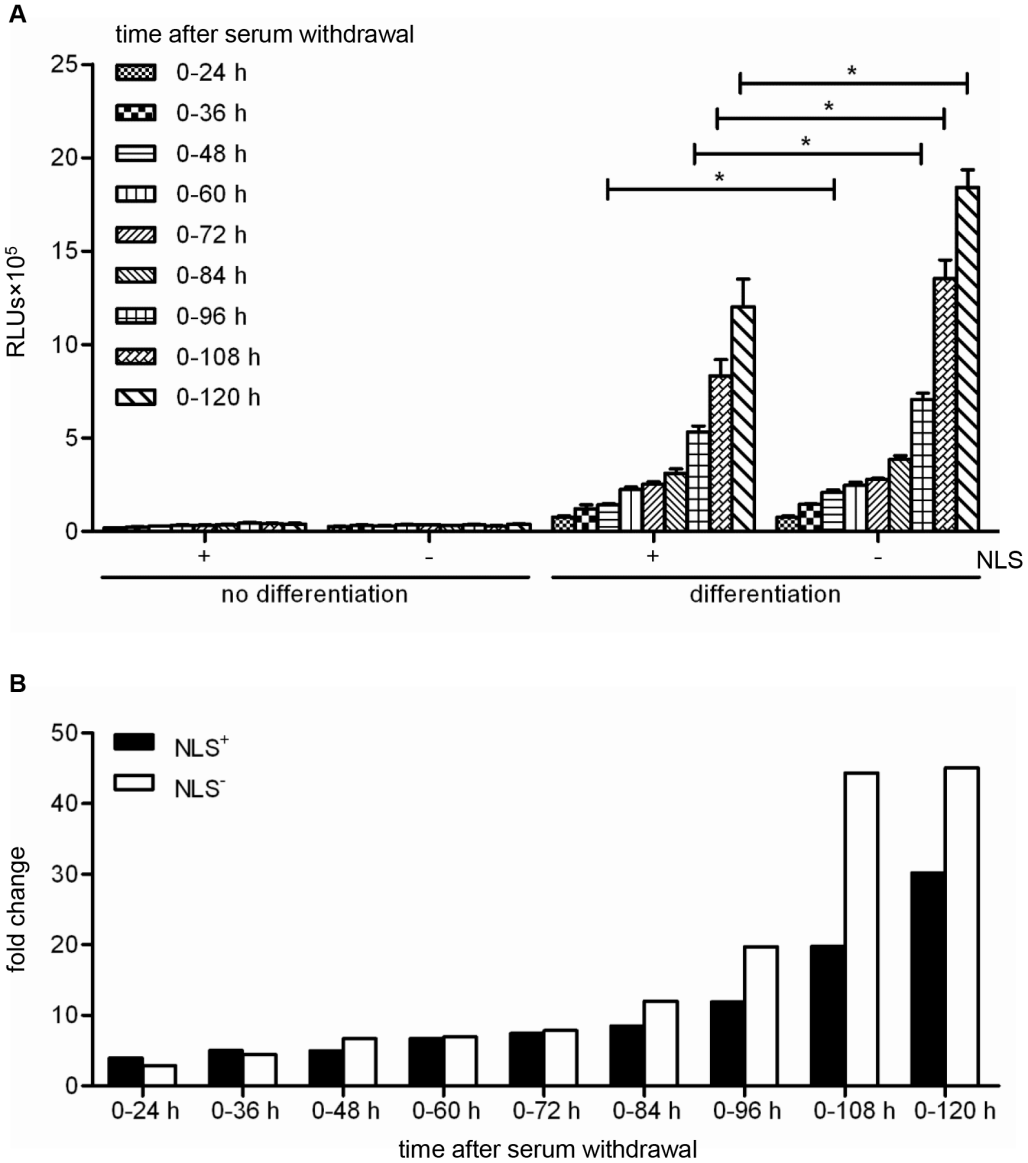
Analysis of GpLuc accumulation in proliferating and differentiating 1∶1 co-cultures of myoblasts^GS.GLuc^ and either myoblasts-FLPe^NLS+^ or myoblasts-FLPe^NLS^^−^. (**A**): Luminometric analysis of culture medium of co-cultures of 50% myoblasts^GS.GLuc^ and 50% myoblasts-FLPe^NLS+^ (+) or 50% myoblasts-FLPe^NLS−^ (−). At 72 h after cell seeding, the culture fluid was replaced by fresh culture medium with (growth conditions, no differentiation) or without (differentiation conditions) serum, which was left on the cells for the indicated periods of time. Bars represent mean ± standard error of the mean (n = 3). (**B**): Fold change in luciferase activity calculated on the basis of the data presented in (A). For each sampling time the average light production under growth conditions was the denominator and the mean of the RLUs produced under differentiation conditions was the numerator. RLUs, relative light units; NLS, nuclear localization signal.

**Figure 9:**
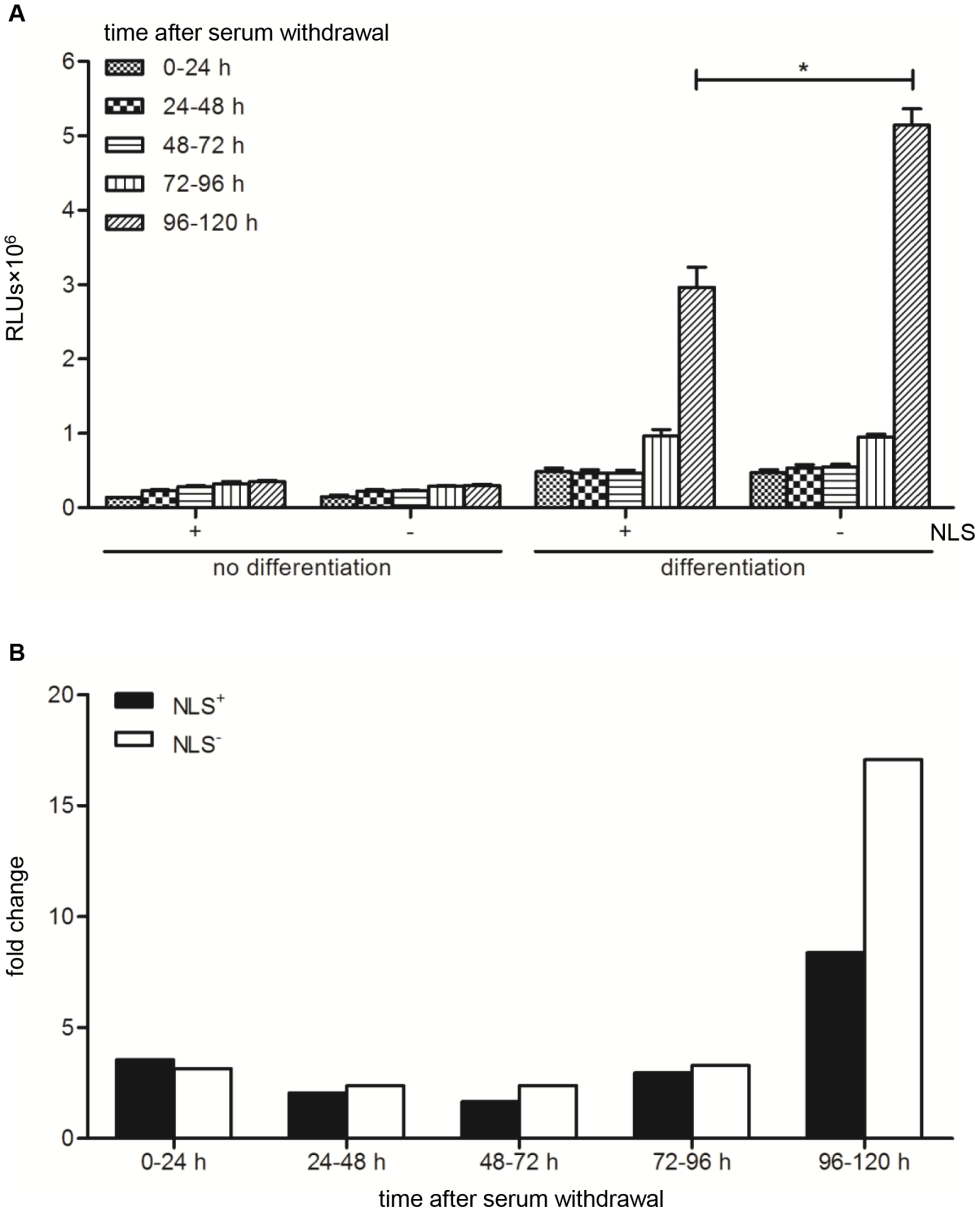
Analysis of GpLuc secretion in proliferating and differentiating 1∶1 co-cultures of myoblasts^GS.GLuc^ and either myoblasts-FLPe^NLS+^ or myoblasts-FLPe^NLS^^−^. (**A**): Luminometric analysis of culture medium of co-cultures of 50% myoblasts^GS.GLuc^ and 50% myoblasts-FLPe^NLS+^ (+) or 50% myoblasts-FLPe^NLS−^ (−). At 72 h after cell seeding, the culture fluid was replaced by fresh culture medium with (growth conditions, no differentiation) or without (differentiation conditions) serum, which was left on the cells for the indicated 24-h time intervals. Bars represent mean ± standard error of the mean (n = 3). (**B**): Fold change in luciferase activity calculated on the basis of the data presented in (A). For each sampling time the average light production under growth conditions was the denominator and the mean of the RLUs produced under differentiation conditions was the numerator. RLUs, relative light units; NLS, nuclear localization signal.

To investigate the utility of the LV.FLPe^NLS+/−^.PurR/LV.GS.GpLuc-based cell fusion assay system to follow cell fusion progression, myoblasts^GS.GLuc^ were mixed with myoblasts-FLPe^NLS+^ or with myoblasts-FLPe^NLS−^ at a ratio of 50∶50. After the cell cultures had become nearly confluent, they were either given fresh growth medium or exposed to differentiation medium. This was followed by the periodic collection of culture fluid for luciferase measurements using two different approaches. In one experiment, the culture medium was left on the cells for different time periods (*i.e.* from 0–24, 0–36, 0–48, 0–60, 0–72, 0–84, 0–96, 0–108 and 0–120 h) before being harvested for luminometry (“cumulative assay”; Fig. 8). In the other experiment, the culture fluid was refreshed every 24 h and the amount of biologically active luciferase that had been secreted between 0–24, 24–48, 48–72, 72–96 and 96–120 h after the start of the differentiation process was determined (“kinetics assay”; Fig. 9). As shown in Fig. 8A, following an initial slow increase, the luciferase activity in the culture medium of the serum-deprived co-cultures rose sharply at late times (>72 h) after initiation of differentiation. Co-cultures of myoblasts^GS.GLuc^ and myoblasts-FLPe^NLS−^ produced better results than the combination of myoblasts^GS.GLuc^ and myoblasts-FLPe^NLS+^ (Fig. 8A,B) in spite of the much higher FLPe concentration in myoblasts-FLPe^NLS+^ than in myoblasts-FLPe^NLS−^ (Fig. 5A). These findings were corroborated by the data derived from the “kinetics assay” (Fig. 9).

.

**Figure 10:**
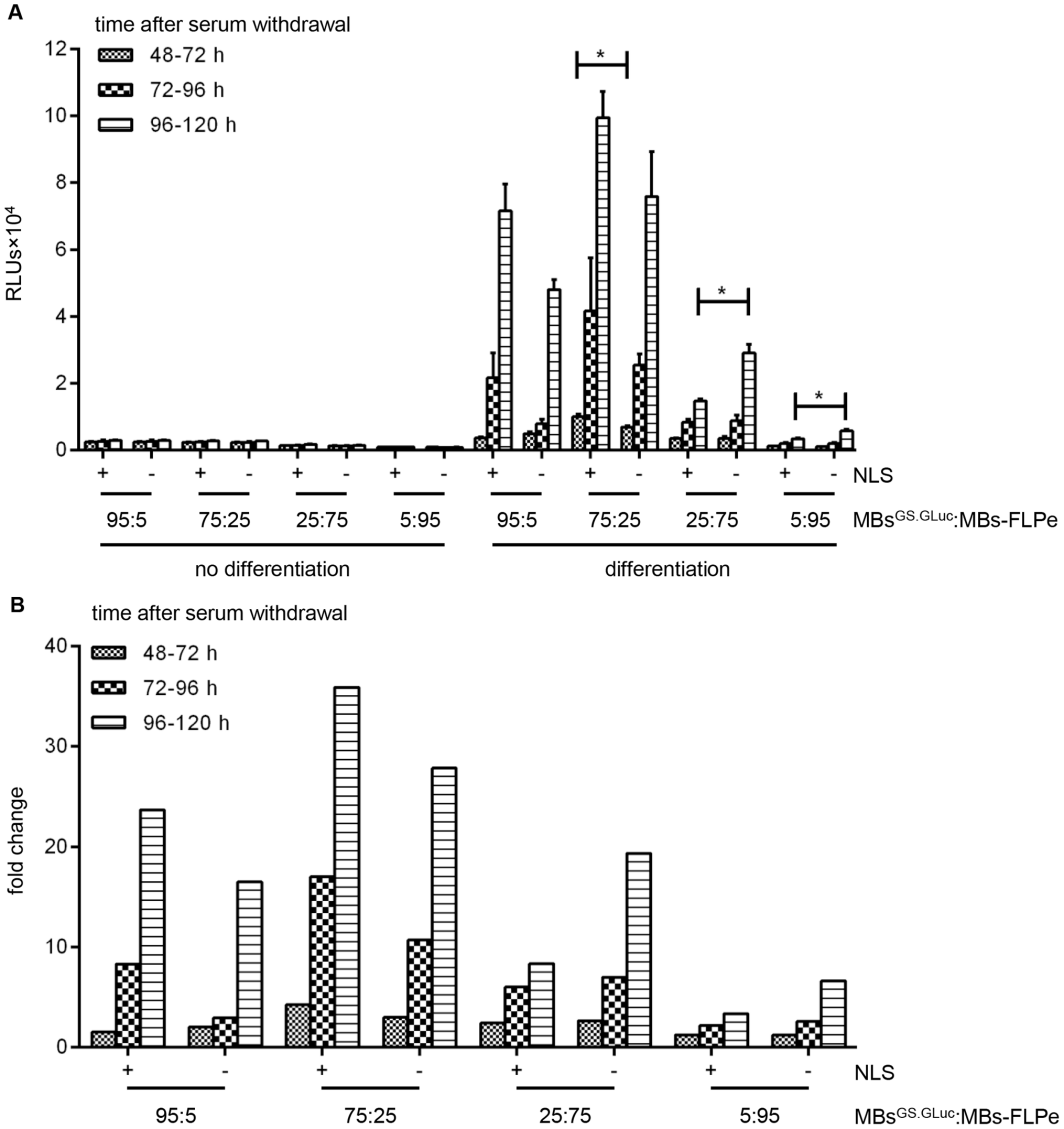
Performance of FLPe^NLS+^ and FLPe^NLS−^ at different acceptor-to-donor cell ratios and time points. (**A**): GpLuc release by proliferating or differentiating co-cultures of myoblasts^GS.GLuc^ and myoblasts-FLPe^NLS+^ (+) or myoblasts-FLPe^NLS−^ (−) at different time intervals after culture initiation. Myoblasts^GS.GLuc^ and myoblasts-FLPe were seeded in different ratios (*i.e.* 95∶5%, 75∶25%, 25∶75% and 5∶95%). At 72 h after cell seeding, the culture fluid was replaced by fresh culture medium with (growth conditions, no differentiation) or without (differentiation conditions) serum. Fourthy-eight h later the culture medium was refreshed once again. Twenty-four h later the culture fluid was harvested for luciferase activity measurement and replaced by the same volume of fresh culture medium. This procedure was repeated every 24 h until 120 h after the first medium change. Bars represent mean ± standard error of the mean (n = 3). (**B**): Fold change in luciferase activity calculated on the basis of the data presented in (A). For each experimental condition the average light production under growth conditions was the denominator and the mean of the RLUs produced under differentiation conditions was the numerator. RLUs, relative light units; MBs^GS.GLuc^, myoblasts^GS.GLuc^; MBs-FLPe, myoblasts-FLPe; NLS, nuclear localization signal.

On the basis of the previous results, another experiment was carried out in which we directly compared the performance of FLPe^NLS+^ and FLPe^NLS−^ at different myoblast^GS.GLuc^:myoblast-FLPe ratios (*i.e.* 95∶5, 75∶25, 25∶75 and 5∶95) and different time points (*i.e.* 72, 96 and 120 h after serum withdrawal). The culture medium was refreshed just before the start of the first sampling interval (*i.e.* at 48 h after serum removal) and after each round of sample collection. This experiment confirmed that at high myoblast^GS.GLuc^:myoblast-FLPe ratios FLPe^NLS−^ was nearly as efficient as FLPe^NLS+^ at inducing reporter gene expression while at low myoblast^GS.GLuc^:myoblast-FLPe ratios FLPe^NLS−^ gave rise to more RLUs (Fig. 10A) and to higher signal-to-noise ratios (Fig. 10B). In accordance with the experiment presented in Fig. 7, the co-cultures consisting of 75% myoblasts^GS.GLuc^ and 25% myoblasts-FLPe yielded the highest signals both in absolute (Fig. 10A) and relative (Fig. 10B) terms. Also in line with the previous experiments was the finding that most GpLuc accumulation takes place between 96 and 120 h after serum removal.

### Comparison of LV.GS.GpLuc.v1- and LV.GS.PpLuc-based cell fusion assay systems

**Figure 11:**
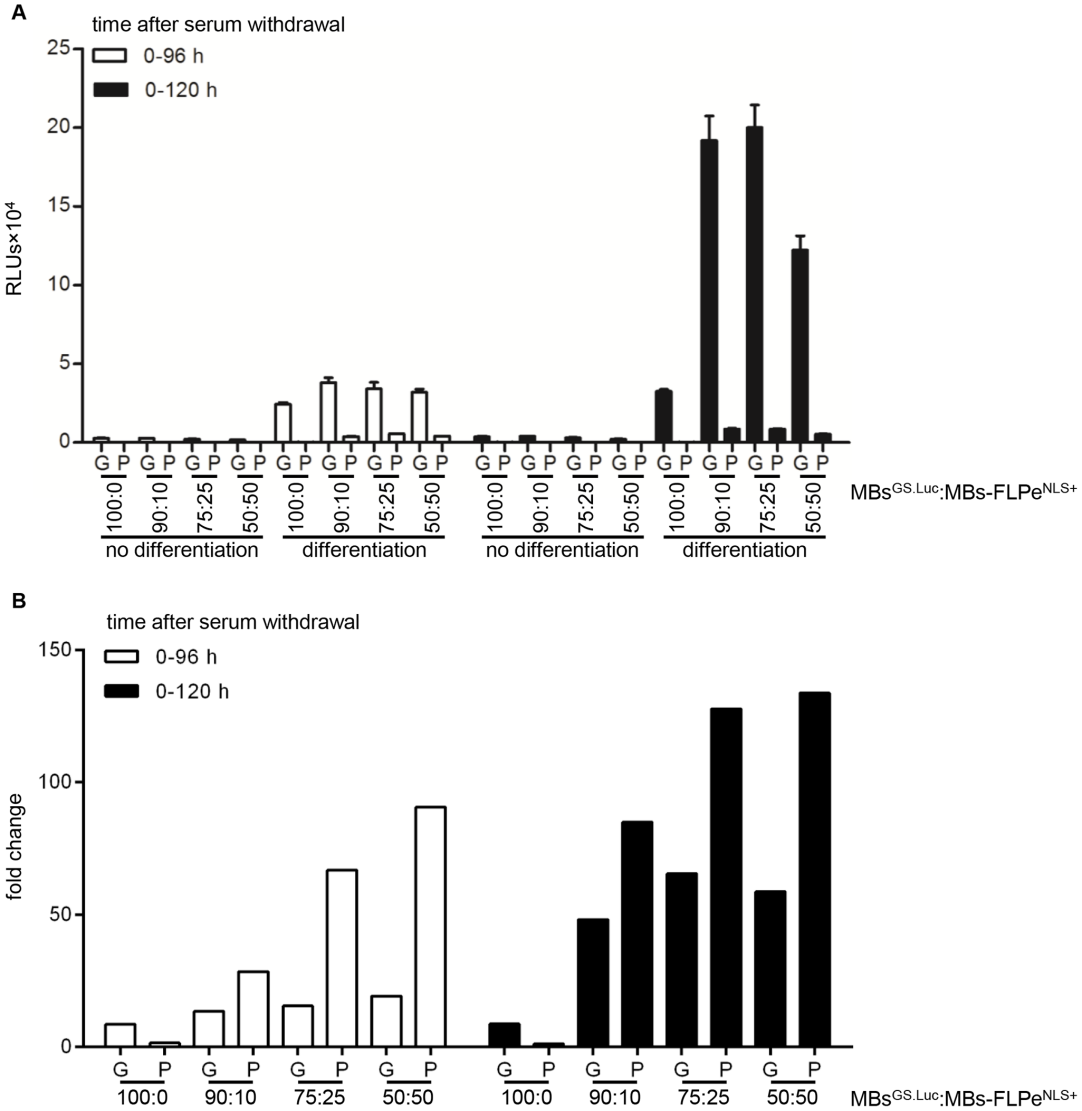
Comparison of LV.GS.GpLuc.v1- and LV.GS.PpLuc-based cell fusion assay systems. (**A**): GpLuc and PpLuc production by proliferating or differentiating co-cultures of myoblasts^GS.GLuc^ or myoblasts^GS.PLuc^ with myoblasts-FLPe^NLS+^ at different times after culture initiation. Cells were seeded in different ratios (*i.e.* 100∶0%, 90∶10%, 75∶25% and 50∶50%). At 72 h after cell seeding the culture fluid was replaced by fresh culture medium with (growth conditions, no differentiation) or without (differentiation conditions) serum. Ninety-six h and 120 h later samples (culture fluid for cultures containing myoblasts^GS.GLuc^ and cell lysates for cultures containing myoblasts^GS.PLuc^) were harvested for luciferase activity measurements. Bars represent mean ± standard error of the mean (n = 3). (**B**): Fold change in luciferase activity calculated on the basis of the data presented in (A). For each experimental condition the average light production under growth conditions was the denominator and the mean of the RLUs produced under differentiation conditions was the numerator. RLUs, relative light units; G, LV.GS.GpLuc.v1-based cell fusion assay; P, LV.GS.PpLuc-based cell fusion assay; MBs^GS.Luc^, myoblasts^GS.GLuc^ or myoblasts^GS.PLuc^; MBs-FLPe^NLS+^, myoblasts-FLPe^NLS+^.

In the next experiment, a direct comparison was made between the previously described LV.GS.PpLuc-based quantitative cell fusion assay system [7] and the new LV.GS.GpLuc-based method to quantify cell-to-cell fusion. Consistent with the much higher light output of GpLuc than of PpLuc [8], LV.GS.GpLuc yielded up to 23-fold higher signals than LV.GS.PpLuc (Fig. 11A). However, the LV.GS.PpLuc-based cell fusion assay system appeared to be approximately twice as sensitive as its LV.GS.GpLuc-based counterpart at detecting myoblast-to-myoblast fusion at 120 h after initiation of differentiation (Fig. 11B). The difference in sensitivity between the GS.GpLuc.v1- and LV.GS.PpLuc-based cell fusion assay systems was even bigger for the samples collected at 96 h after serum removal especially at the lowest two myoblast^GS.Luc^:myoblast-FLPe^NLS+^ ratios (*i.e.* when FLPe levels are highest).

### Improvement of the GS.GpLuc-based cell fusion assay system

The results presented in Figs. 7 and 11 identify the FLP-independent increase in GpLuc production when shifting from growth to differentiation medium as the main contributor to the reduced signal-to-noise ratio of the LV.GS.GpLuc-based cell fusion assay system as compared to its LV.GS.PpLuc-based counterpart. In search for a possible explanation for the high background signal produced by LV.GS.GpLuc.v1 in comparison to LV.GS.PpLuc, we compared their genetic organization upstream of the *Luc* start codon. As shown in Figs. 2 A,B and 3 the *PpLuc* ORF in LV.GS.PpLuc is preceded by an out-of-frame ORF (uORF) starting with 2 ATG codons in a favourable context for translational initiation [19] and ending with a highly efficient stop codon [20] separated by only 7 nucleotides from the *PpLuc* initiation codon. This specific genetic makeup will be effective in supressing any *PpLuc* expression directed by mRNAs with 5′ ends located upstream of the second ATG codon in the uORF. Oppositely, in LV.GS.GpLuc.v1 the previously mentioned tandem of ATG codons are in-frame with the *GpLuc* initiation codon allowing the synthesis of an N-terminally extended GpLuc fusion protein. Located further upstream of the *GpLuc* ORF in LV.GS.GpLuc.v1 is an out-of-frame ORF with suboptimal start and stop codons. LV.GS.GpLuc.v1 thus offers much more possibilities for “leaky” *Luc* expression than LV.GS.PpLuc. To solve this problem, we designed LV.GS.GpLuc.v6. In this construct, the distance between the mMT1 pA and *GpLuc* ORF is kept very short to minimize the chance of creating transcriptional start sites in the intervening region. As an additional measure to limit leaky *GpLuc* expression, LV.GS.GpLuc.v6 contains a 21-bp uORF starting immediately upstream of the FRT sequence and ending with an efficient stop codon provided by the FRT sequence. Between the stop codon of the uORF and the *PpLuc* initiation codon only 20 nucleotides are present comprising the remainder of the FRT sequence and an optimal start site for *GpLuc* translation.

**Figure 12:**
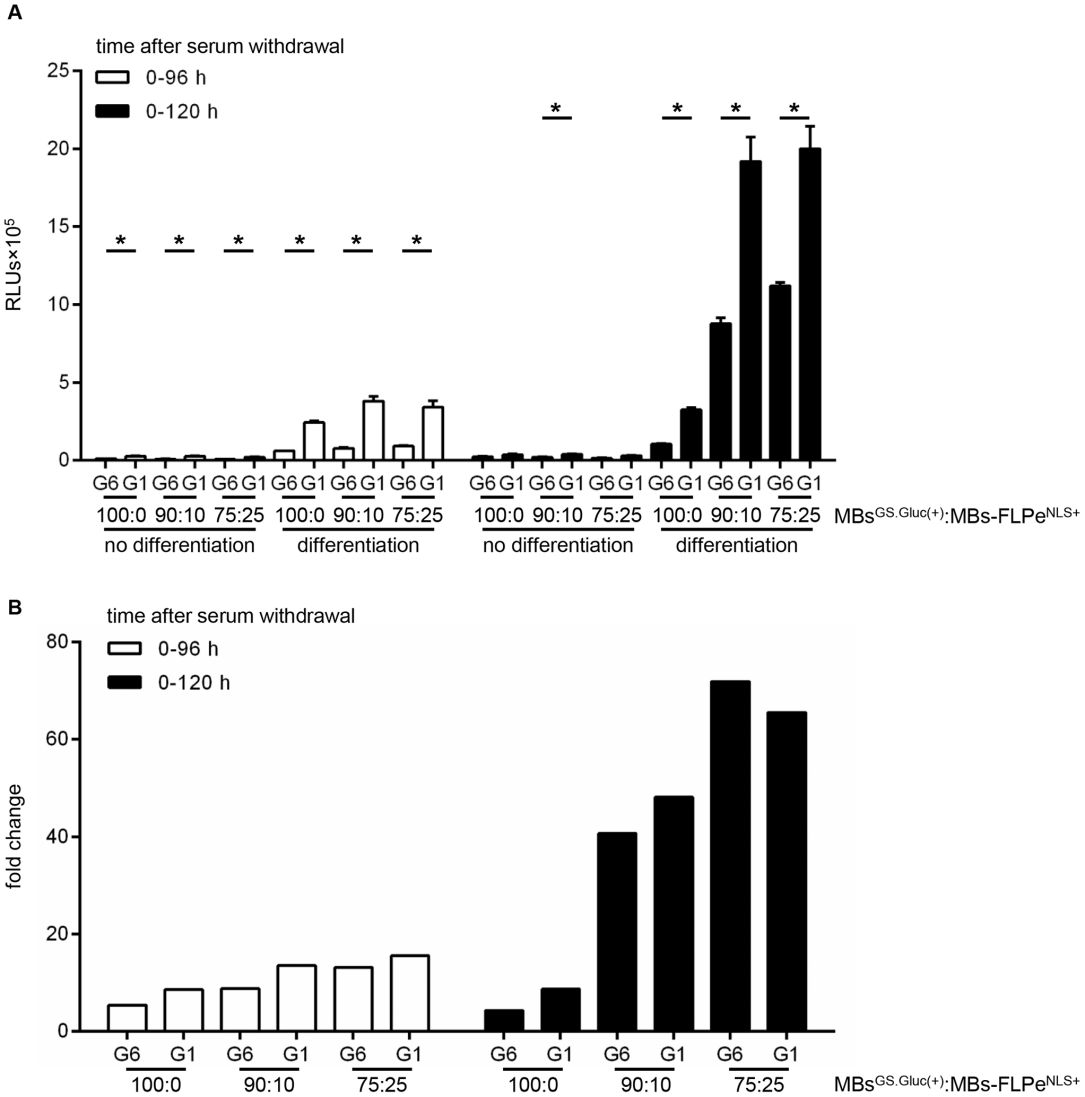
Comparison of LV.GS.GpLuc.v1- and LV.GS.GpLuc.v6-based cell fusion assay systems. (**A**): GpLuc production by proliferating or differentiating co-cultures of myoblasts^GS.GLuc^ or myoblasts^GS.GLuc+^ with myoblasts-FLPe^NLS+^ at different times after culture initiation. Cells were seeded in different ratios (*i.e.* 100∶0%, 90∶10% and 75∶25%). At 72 h after cell seeding the culture fluid was replaced by fresh culture medium with (growth conditions, no differentiation) or without (differentiation conditions) serum. Ninety-six h and 120 h later culture medium collected for luciferase activity measurement. Bars represent mean ± standard error of the mean (n = 3). (**B**): Fold change in luciferase activity calculated on the basis of the data presented in (A). For each experimental condition the average light production under growth conditions was the denominator and the mean of the RLUs produced under differentiation conditions was the numerator. RLUs, relative light units; G1, LV.GS.GpLuc.v1-based cell fusion assay; G6, LV.GS.GpLuc.v6-based cell fusion assay; MBs^GS.GLuc(+)^, myoblasts^GS.GLuc^ or myoblasts^GS.GLuc+^; MBs-FLPe^NLS+^, myoblasts-FLPe^NLS+^.

LV.GS.GpLuc.v6 was used to generate myoblasts^GS.GLuc+^ carrying the optimized *GpLuc* gene switch cassette. Next, the performance of the LV.GS.GpLuc.v1- and LV.GS.GpLuc.v6-based cell fusion assay systems was compared in an experiment with the same setup as used for the comparison of LV.GS.GpLuc.v1 with LV.GS.PpLuc except for the omission of the 1∶1 myoblast^GS.GLuc(+)^:myoblast-FLPe^NLS+^ ratio. Luciferase activity in 0–96 h and 0–120 h culture medium of serum-deprived myoblast^GS.GLuc+^ monocultures was ±3-fold lower than in culture medium of differentiating myoblast^GS.GLuc^ monocultures (Fig. 12), demonstrating the effectiveness of the new gene switch design to inhibit leaky *GpLuc* expression. However, since the improved gene switch design also reduced FLPe-dependent signal output the fold increase in GpLuc activity during myogenic differentiation of myoblast^GS.GLuc(+)^:myoblast-FLPe^NLS+^ co-cultures was quite similar for LV.GS.GpLuc.v1 and LV.GS.GpLuc.v6 (Fig. 12B). Still, in comparison to LV.GS.GpLuc.v1 for LV.GS.GpLuc.v6 a much larger part of the increase in GpLuc activity observed in differentiating myoblast^GS.GLuc(+)^:myoblast-FLPe^NLS+^ co-cultures is attributable to cell fusion.

## Discussion

Apart from being involved in the formation and maintenance of skeletal muscles, bones and the placenta, cell-to-cell fusion plays an important role in numerous other biological processes like fertilization. It has also been implicated in the initiation and progression of cancer [2] and as a driving force in evolution [21]. Moreover, cell-to-cell fusion has been of great value to establish the chromosomal location of specific genes [22], can be used to induce cellular reprogramming [23], [24] and is indispensable for generating hybridomas [25]. The involvement of cell-to-cell fusion in a large variety of biological processes and its diverse biotechnological applications have prompted investigations into the mechanisms of cell fusion and the contribution of specific factors to this process. Instrumental to this research is the availability of robust assays to determine cell fusion kinetics and extent. However, most of the existing quantitative cell fusion assays do not allow consecutive analysis of the same cells/tissue. Accordingly, in this paper a new quantitative assay is presented to monitor cell-to-cell fusion. This assay is based on the activation of a latent *GpLuc* gene after fusion of cells containing this latent reporter gene with cells encoding a recombinase that activates the dormant *GpLuc* gene. The extent of cell-to-cell fusion is subsequently quantified by simply measuring the enzymatic activity of the luciferase molecules secreted by the cellular fusion products. To the best of our knowledge this is the first assay that allows quantification of cell fusion activity by medium sampling.

To validate the new cell fusion assay it was used to monitor the formation of myotubes/sacs in cultures of serum-deprived human myoblasts. In these experiments, several parameters were varied including the acceptor-to-donor cell ratio and the sample regimen(s) of the cell culture medium. In general, transgene expression increased with increasing fractions of myoblasts^GS.GLuc^ up to the point at which the number of active/nuclear FLPe molecules became limiting (*i.e.* at myoblast^GS.GLuc^:myoblast-FLPe ratios of 90∶10 for FLPe^NLS−^ and of 95∶5 for FLPe^NLS+^; Fig. 7).

At high myoblast^GS.GLuc^:myoblast-FLPe ratios LV.FLPe^NLS+^ was slightly more effective than LV.FLPe^NLS−^ in activating the latent *GpLuc* gene most likely due to fact that under differentiation conditions myoblasts-FLPe^NLS+^ contain ±5-fold more nuclear FLPe molecules than myoblasts-FLPe^NLS−^ (Fig. 5A). In contrast, at low myoblast^GS.GLuc^:myoblast-FLPe ratios (*i.e.* when FLPe is no longer limiting) LV.FLPe^NLS−^ consistently outperformed LV.FLPe^NLS+^ (Figs. 7 and 10). Collectively, these findings suggest that its NLS does not noticeably hamper the spreading of FLPe^NLS+^ through myofibers/sacs but that high nuclear FLPe levels may somehow limit reporter gene expression. A possible explanation for the higher *GpLuc* expression in differentiating co-cultures containing large percentages of myoblasts-FLPe^NLS−^ in comparison to those with large fractions of myoblasts-FLPe^NLS+^ may be the more frequent occurrence of secondary recombination events in the latter co-cultures leading to the deactivation of functional *GpLuc* expression modules.

While monocultures of myoblasts^GS.GLuc^ maintained in growth medium displayed very little if any leaky *GpLuc* expression, considerable amounts of GpLuc were produced by myoblast^GS.GLuc^ monocultures exposed to differentiation medium. There are several possible explanations for this finding. Firstly, growth and differentiation medium may differently affect light output *e.g.* by (i) causing different levels of coelenterazine “auto-oxidation”, (ii) containing different concentrations of chemiluminescence inhibitors or (iii) absorbing blue light to a different extent. Possibilities (i) and (iii) can be ruled out since mixing of coelenterazine substrate solution with fresh or myoblasts-FLPe^NLS+^-conditioned growth or differentiation medium produced very similar signals (data not shown). This leaves us with the possibility that transcription termination by the mMT1 pA incorporated into the gene switch constructs is not very efficient or that differentiation conditions somehow stimulate transcription initiation in the region located in between the mMT1 pA and the *Luc* ORFs. For LV.GS.PpLuc and LV.GS.GpLuc.v6 the resulting transcripts may not lead to substantial luciferase production due to the presence of “decoy” ORFs immediately upstream of the *Luc* initiation codons (Figs. 2 and 3). A similar favorable situation does not exist for LV.GS.GpLuc.v1, which may explain the high background signals produced by this construct under differentiation conditions. Even though the luciferase activity in culture medium of differentiating myoblast^GS.GLuc+^ monocultures is ±3-fold lower than in culture medium of differentiating myoblast^GS.GLuc^ monocultures LV.GS.GpLuc.v6 still gives rise to a higher background signal under differentiation conditions than LV.GS.PpLuc (compare Fig. 11 with 12). Considering that the sequences in between the mMT1 pA and the *Luc* start codon in LV.GS.PpLuc and LV.GS.GpLuc.v1 are nearly identical this may suggest that the GpLuc-coding sequence itself is the source of the relatively high luciferase activity detected in medium of differentiating LV.GS.GpLuc monocultures. If so, the problem could be overcome by switching to another secretory luciferase (*e.g. Vargula hilgendorfii* luciferase [26], Lucia luciferase (InvivoGen Europe, Toulouse, France) or secretory NanoLuc [27]). Also the fact that GpLuc is a secretory protein with a long half-life (±6 days in culture medium) [28] while Ppluc has a relatively short half-life (±2 hours in cells) [29] may contribute to the higher background signals associated with LV.GS.GpLuc.v1 and LV.GS.GpLuc.v6 than with LV.GS.PpLuc.

Taken together, in this paper a new assay to quantify (the progression of) cell-to-cell fusion activity is described. Due to its nondestructive nature allowing repeated sampling of the same specimen, this assay will be an attractive alternative to existing quantitative cell fusion assays based on (i) light microscopic assessment of multinucleation, (ii) fluorescence dequenching, (iii) fluorescence resonance energy transfer, (iv) biochemical complementation or (v) activation of reporter genes different from *GpLuc* including *LacZ* and *PpLuc* [3]. Other advantages of the LV.FLPe^NLS+/−^.PurR/LV.GS.GpLuc-based cell fusion assay include the simplicity and speed of the analytical procedures and the ability to combine it with (immuno)cytology, real-time microscopy, cell function assays and other methods to study cell behavior.

The sensitivity of the current assay could be improved by changing the human *glyceraldehyde 3-phosphate dehydrogenase* (*hGAPDH*) gene promoter driving *GpLuc* expression for a promoter with higher activity in the cell type(s) under investigation. In addition, the sequences interspersed between the 3′ long terminal repeat (LTR) and the *GpLuc* initiation codon of LV.GS.GpLuc.v6 may be further optimized to minimize leaky *GpLuc* expression.
